# Integrating Structural, Dielectric and Mechanical Properties to Evaluate the Performance of NR/SBR/GTR/SiO_2_ Compounds

**DOI:** 10.3390/polym18121448

**Published:** 2026-06-10

**Authors:** Ramon Mujal-Rosas, Miguel Mudarra-Lopez, Marc Marín-Genescà, Manuel Lis Arias, Xavier Colom

**Affiliations:** 1 Departament d’Enginyeria Eléctrica, ESEIAAT-UPC, Colom, 1, 08222 Terrassa, Catalonia, Spain; ramon.mujal@upc.edu; 2 Departament de Física, ESEIAAT-UPC, Colom, 1, 08222 Terrassa, Catalonia, Spain; miguel.mudarra@upc.edu; 3 Departament d’Enginyeria Mecànica, ETSEQ-URV, Països Catalans nº 26, 43007 Tarragona, Catalonia, Spain; 4 Departament d’Enginyeria Química, ESEIAAT-UPC, Colom, 1, 08222 Terrassa, Catalonia, Spain; manuel-jose.lis@upc.edu (M.L.A.);

**Keywords:** devulcanized ground tire rubber, NR/SBR compounds, interfacial polarization, dielectric spectroscopy, movement conduction, structure–property relationships

## Abstract

The incorporation of ground tire rubber (GTR) into elastomeric compounds offers a sustainable route for recycling end-of-life tires; however, its effect on the structure–property relationships governing mechanical and dielectric performance remains insufficiently understood. In this study, NR/SBR composites containing 0–50 phr of devulcanized GTR were prepared and characterized through Fourier-transform infrared spectroscopy (FTIR), swelling analysis, thermogravimetric analysis (TGA), mechanical testing, and broadband dielectric spectroscopy. FTIR and swelling results revealed enhanced matrix–GTR interaction at intermediate GTR loadings (10–20 phr), evidenced by an increased intensity of sulfur-related bands and reduced swelling degree, indicating partial chemical integration of the recycled phase into the elastomer network. Mechanical testing showed that increasing GTR content increased stiffness at high loadings, while tensile strength, elongation at break, and toughness progressively decreased due to interfacial debonding mechanisms. TGA demonstrated that the main degradation temperature of the NR/SBR matrix remained essentially unchanged (418–425 °C) across all formulations, confirming preservation of thermal stability despite increasing structural heterogeneity. Dielectric spectroscopy (10^−2^–3 × 10^6^ Hz, 40–120 °C) revealed pronounced Maxwell–Wagner–Sillars interfacial polarization and thermally activated charge transport, with conductivity increasing with GTR content without evidence of electrical percolation, even at 50 phr. The results demonstrate that the performance of NR/SBR/GTR/SiO_2_ composites is primarily controlled by the interfacial structure generated by the recycled phase. Intermediate GTR contents (10–20 phr) provide the most effective matrix–GTR interaction, while higher loadings mainly affect mechanical integrity and dielectric response through increased structural heterogeneity. These findings provide practical guidelines for designing sustainable elastomeric compounds with high recycled content while maintaining thermal stability and controlled electrical insulation properties.

## 1. Introduction

The valorization of end-of-life tires represents a significant technological and environmental challenge due to the high structural stability of vulcanized elastomers. Although this stability ensures excellent mechanical durability during service, it also hinders efficient recycling and reuse. Among the strategies proposed to address this problem, the use of ground tire rubber (GTR) obtained from mechanical grinding of waste tires has attracted considerable attention as a sustainable secondary raw material for polymer compounds [1].

However, the direct incorporation of GTR into virgin elastomer matrices often leads to a deterioration of mechanical properties because the recycled particles remain highly crosslinked and exhibit limited compatibility with the surrounding polymer network [2]. To overcome this limitation, different devulcanization processes have been widely investigated as a method to partially break sulfur crosslinks and restore chain mobility, enabling the recycled material to be reprocessed and blended with fresh elastomers such as natural rubber (NR) or styrene–butadiene rubber (SBR) [3].

Despite these advances, compounds containing devulcanized GTR remain intrinsically heterogeneous systems formed of partially crosslinked rubber domains, filler particles and interfacial regions with different structural characteristics. Several studies have demonstrated that this multiphase arrangement strongly influences the thermal stability, microstructure and mechanical behavior of recycled rubber compounds [4]. In particular, the presence of recycled domains introduces discontinuities in the polymer network that modify stress transfer mechanisms and can promote interfacial debonding phenomena at high recycled contents [5].

While several investigations have focused primarily on mechanical or thermal performance, the relationship between interfacial heterogeneity and electrical charge transport in recycled elastomer compounds has received less attention. Broadband dielectric spectroscopy represents a powerful technique for studying heterogeneous polymer systems because it allows for the identification of interfacial polarization processes, space-charge accumulation and thermally activated hopping transport mechanisms over a wide frequency and temperature range [6].

In general, silica acts as a reinforcing filler that increases the stiffness and hardness of elastomer compounds while maintaining their insulating character.

In this context, the present work investigates NR/SBR compounds containing increasing amounts of devulcanized GTR (0–50 phr) through an integrated thermal, mechanical and dielectric characterization approach. The aim is to establish a structure–property correlation that clarifies how the recycled phase modifies the interfacial structure and consequently affects thermal stability, mechanical integrity and electrical connectivity. Such an integrated approach contributes to the development of sustainable elastomeric materials with high recycled content while preserving their functional performance.

## 2. Materials and Methods

### 2.1. Materials

The virgin elastomers employed in this study, supplied by VIGAR HEXPOL Compounding S.L.U. (Rubí, Spain), consisted of natural rubber (NR) and styrene–butadiene rubber (SBR). Specifically, the NR used was of the SVR CV type (originally from Ho Chi Minh, Vietnam), characterized by a density of 0.92 g/cm^3^, a plasticity retention index (PRI) of 60, and a low impurity content (maximum 0.4 wt% ash and 0.8 wt% volatile matter). The SBR grade selected was Pliogum 1027K1, featuring 24 wt% bound styrene and a Mooney viscosity (ML1+4 at 100 °C) of 52.

The ground tire rubber (GTR) was provided by Gestión Medioambiental de Neumáticos S.L. (GMN) in Maials, Spain. This material originates from the recycling of a mixture of passenger car and truck tires, exhibiting a maximum particle size of 0.7 mm and an average particle diameter of 320 µm. Detailed particle size distribution can be found in studies reported by Li et al. [7]. Thermogravimetric analysis (TGA) performed on the samples revealed a composition of 29% NR, 31% synthetic rubber, 29.5% carbon black, 7% SiO_2_, and 3.5% other additives (such as processing aids and curing agents), values that are consistent with the results [7].

The additives and curing agents used for curing, namely vulcanization accelerators (TBBS N-cyclohexil-2-benzotiazolsulfenamide, TMTD-tetramethylthiuram disulfide), carbon black N550, stearic acid, zinc oxide, sulfur with technical purity, and SiO_2_ (85% particle size below 150 µm), were supplied by VIGAR (Rubí, Spain). Bis (3-triethoxysilyl propyl) tetrasulfide (C_18_H_42_O_6_S_4_Si_2_; TESPT) activated the devulcanization. It was also supplied by VIGAR (Rubí, Spain).

### 2.2. Devulcanization of GTR

The GTR devulcanization process was conducted via a combined thermo-mechanical and chemical protocol, a methodology previously reported in the literature [8]. Specifically, the treatment involves processing the GTR with the addition of 2 parts per hundred rubber (phr) of TESPT, which functions as a devulcanizing agent [3]. This mixture was subjected to 10 passes through the equipment under constant operating conditions of 50 °C and 80 rpm. Upon completion of the treatment, the resulting material exhibited a putty-like consistency, characteristic of the devulcanized state.

### 2.3. Preparation of Rubber Compounds

The rubber compounds were formulated according to the specifications detailed in Table 1. The compounding process was executed in three distinct stages using a Brabender Plasticorder internal mixer (Brabender GmbH & Co. KG, Duisburg, Germany) set to 80 °C and 80 rpm. Initially, the virgin matrix (NR and SBR) underwent a 5 min plasticization period. Subsequently, the reinforcing fillers (30 phr carbon black and 30 phr SiO_2_) were incorporated together with the devulcanized GTR (dGTR) and mixed for an additional 5 min.

The process concluded with the addition of the sulfur curing system (consisting of 5.0 phr ZnO, 3.0 phr stearic acid, 1.0 phr TBBS, 0.25 phr TMTD, and 2.0 phr sulfur), which was mixed for 2 min to ensure proper dispersion without initiating premature vulcanization. The formulation was designed to replicate a standard tire composition while integrating varying levels of dGTR (0, 10, 20, 40 and 50 phr). To maintain a constant total filler content, the amount of fresh silica and carbon black added was adjusted based on the GTR loading. Since 10 phr of dGTR contributes approximately 0.7 phr of silica and 3 phr of carbon black, the addition of fresh fillers was reduced proportionally for each increment of recycled material. The curing package remained constant across all formulations.

To ensure homogeneity, two batches from the internal mixer were combined using a Collin Teach-line two-roll mill. The resulting material was compression-molded in a Collin P 200E hot plate press at 160 °C and 200 bar. The curing time was set to 12 min [9], corresponding to the optimum cure time (t90) determined via a Monsanto Oscillating Disc Rheometer R 100 (Akron, OH, USA) at 162 ± 1 °C, following ASTM D2084 standards [10]. Upon completion, the molds were rapidly cooled, and the vulcanized plates were die-cut into dumbbell-shaped specimens using a J.BOT Instruments cutter (Barcelona, Spain), in accordance with ASTM D-412 [11].

### 2.4. Mechanical Analysis

Stress–strain tests were carried out using an Instron 3366–2 kN universal testing machine (Instron, High Wycombe, UK) following ASTM D412 Die C specifications. The crosshead speed was 20 mm/min. Tests were performed at 23 ± 2 °C and 50 ± 5% relative humidity.

The mechanical characterization included Young’s modulus, tensile strength, elongation at break and toughness as a function of the GTR content in the composite matrix [12]. Five specimens were tested for each formulation, and the mean values and standard deviations were calculated.

The swelling degree was determined by immersing ~0.5 g specimens (m_0_) in toluene at room temperature for 72 h to reach equilibrium. After removing excess surface solvent with filter paper, the samples were reweighed (m_t_). Swelling degree was calculated as the percentage mass increase relative to the initial weight. Lower values indicate a higher crosslink density, meaning stronger chemical integration of GTR particles within the elastomeric matrix, which restricts solvent penetration into the polymer network.

### 2.5. FTIR Analysis

The chemical structure of the specimens was characterized using Fourier-transform infrared spectroscopy in attenuated total reflectance mode (FTIR-ATR). Measurements were conducted on a Perkin Elmer Spectrum Two spectrometer (Shelton, CT, USA) equipped with a diamond crystal ATR accessory. Data were collected over a spectral range of 500–3500 cm^−1^ at a resolution of 2 cm^−1^, with 40 scans averaged per spectrum.

### 2.6. Thermal Analysis

Differential scanning calorimetry (DSC) measurements were performed using a Perkin Elmer TGA 8000 apparatus (Shelton, CT, USA). Samples of approximately 10 mg were placed in aluminum pans and analyzed under an oxygen atmosphere.

Non-isothermal experiments were carried out in the temperature range from −50 °C to 250 °C using a heating rate of 10 °C/min. DSC analysis was used to evaluate possible changes in the thermal transitions of the polymer matrix induced by the incorporation of GTR, particularly through the determination of glass transition temperatures (Tg) [13].

### 2.7. Scanning Electron Microscopy

The morphology of samples was observed with a JEOL 5610 scanning electron microscope (Akishima, Japan). Before observation, the samples were covered with a fine gold–palladium layer to increase their conductivity in a vacuum chamber.

### 2.8. Dynamic Electric Analysis

Dielectric parameters were measured using dynamic electrical analysis (DEA) with a BDS 40 broadband dielectric spectrometer (Novocontrol Technologies, Montabaur, Germany) equipped with a Novotherm temperature control system. Measurements were performed using a parallel-plate sensor with compression-molded samples of 25 mm diameter. The dielectric permittivity (ε′), dielectric loss (ε″) and electrical conductivity (σ) were recorded over the frequency range 10^−2^–3 × 10^6^ Hz and within the temperature interval 40–120 °C.

During the measurement, the sample was placed between two electrodes and subjected to an alternating electric field. The resulting electrical response consists of resistive and reactive current components, whose phase difference allows for the determination of the complex dielectric parameters, including real permittivity, imaginary permittivity, phase angle and electrical conductivity.

From the dielectric spectra, additional parameters were derived for further analysis, including the electric modulus formalism (M′ and M″), the DC conductivity (σ_DC_), the crossover frequency (ωp) between DC and dispersive AC regimes, and the sublinear exponent (*n*) obtained from Jonscher’s power law [14]. The dimensions of the specimens were defined according to ASTM D150; samples were cylindrical with 25 mm diameter and 1 mm thickness.

## 3. Results and Discussion

### 3.1. Mechanical and Physical Characterization

Table 2 shows the mechanical and physical results, including the swelling degree, corresponding to the GTR/elastomeric compounds. It is observed how the incorporation of devulcanized GTR generates structural modifications within the continuous and cohesive elastomeric network in a heterogeneous system defined by interfacial discontinuities. The reference compound without recycled content (0 phr GTR) exhibits the behavior of a well-vulcanized NR/SBR crosslinked blend [13], characterized by high deformation (elongation at break of 1050%) and high tensile strength (18.7 MPa), indicating an efficient stress transfer within a homogeneous crosslinked matrix.

The addition of 10 phr GTR produces a significant reduction in tensile strength (13.4 MPa) and elongation at break (712%), with a slight increase in Young’s modulus. As determined by Colom et al. [15], this behavior reflects the presence of partially crosslinked domains that act as rigid inclusions within the continuous matrix. Although these domains allow for constraints that locally increase stiffness, they also break the continuity of the polymer network, reducing the material’s ability to withstand large homogeneous deformations.

In formulations with moderate GTR contents (20 phr), the elastic modulus shows a slight decrease compared to 10 phr, indicating the competition between two differing mechanisms: (i) the stiffening effect of the pre-crosslinked domains and (ii) the reduction in the effective crosslink density of the virgin matrix due to structural discontinuities due to the presence of the GTR domains. The obtained properties demonstrate that the loss of network continuity seems to prevail over the reinforcing effect.

For higher GTR contents (40–50 phr), the Young’s modulus increases progressively up to 2.38 MPa at 50 phr, indicating that the mechanical response is increasingly dominated by the stiff GTR phase. In this range, the system tends towards a structure dominated by the GTR reinforcement, where the partially crosslinked fraction contributes significantly to macroscopic stiffness. However, the tensile strength continues to decrease (11.9 MPa at 50 phr) and the elongation at break stabilizes at around 690–700%, confirming that the increase in stiffness does not translate into an improvement in the resilience of the composite.

The progressive reduction in toughness reinforces this explanation. The material’s ability to absorb mechanical energy decreases with increasing GTR content, indicating a transition in the failure mechanism from cohesive fracture of the elastomeric matrix to processes dominated by weak GTR–elastomeric matrix interaction. The absence of significant chemical changes in the FTIR analysis, as we will see below, suggests that the observed mechanical deterioration is predominantly structural rather than chemical.

In general, the mechanical performance of NR/SBR-GTR compounds is mainly defined by the interfacial structure generated by the GTR [6,13] and less by its content. The GTR acts simultaneously as a stiffening phase and as a source of stress concentration, establishing a compromise between stiffness and ductility controlled by the continuity of the elastomeric network and the quality of the interfacial adhesion.

### 3.2. Structural Characterization by FTIR-ATR

**Figure 1:**
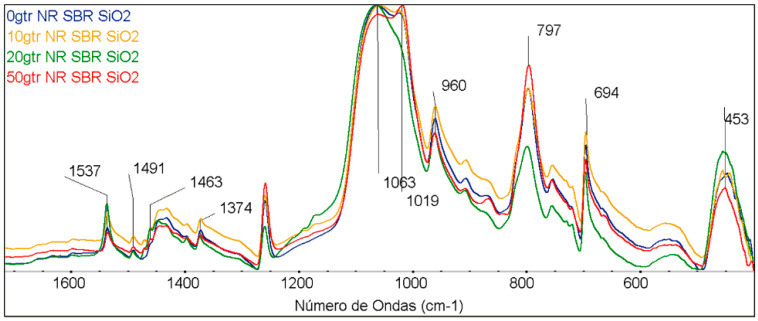
Structural characterization of NR/SBR–GTR compounds as a function of GTR content (0–50 phr).

In Figure 1, the different spectra corresponding to the NR/SBR/GTR/SiO_2_ samples are compared. The typical bands of these compounds highly referenced in different published articles are observed [16,17]. The spectra do not show new bands characteristic of the formation of chemical bonds, confirming that the common interaction that takes place between the elastomeric matrix and GTR is mechanical entanglements [18]. However, a significant increase in the 435 cm^−1^ band (assigned to S-S bonds) is observed in the samples with contents of 10 and 20 phr GTR. This indicates that in compounds with intermediate GTR contents, there is crosslinking formation between the elastomeric matrix and the GTR dispersed in this matrix. In Table 2, where the values of swelling degree are shown, it is observed that the samples with 10 and 20 phr GTR present lower values, which corroborates the results obtained with FTIR. It can be stated that contents of 20 phr of GTR are structurally active and are chemically integrated within the elastomeric matrix.

According to Figure 1, Table 3 shows the most significant bands of NR/GTR composites [18,19,20], so the table shows the relevant wavenumber associated with the different components found in the mix.

### 3.3. Thermal Characterization

**Figure 2:**
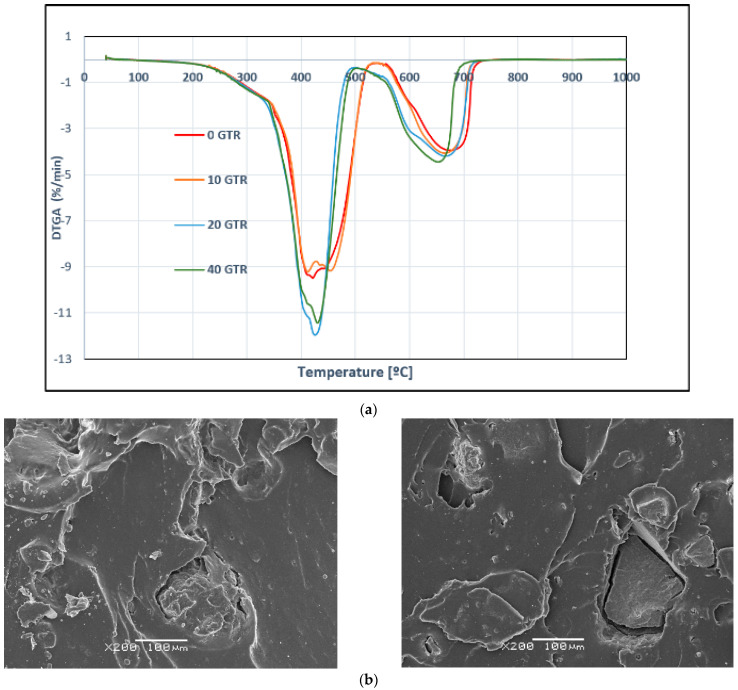
(**a**) TGA and DTG curves of NR/SBR–GTR compounds with increasing recycled content (0–50 phr) measured under a nitrogen atmosphere. The main degradation temperature remains nearly constant, while degradation kinetics change with increasing GTR content. (**b**) SEM micrographs of the NR/SBR/GTR/SiO_2_ compounds with 10 and 50 phr GTR.

The thermogravimetric behavior of NR/SBR/GTR/SiO_2_ compounds containing increasing amounts of GTR, as observed in Figure 2a, was evaluated in an oxidizing atmosphere. Two main decomposition stages were observed for the different formulations. The first stage, associated with the thermo-oxidative decomposition of the elastomeric fraction, takes place between 418 and 425 °C, with variations below 5 °C even at 40 phr GTR. This stability in the maximum thermal decomposition temperature suggests that the incorporation of recycled rubber does not significantly modify the thermal behavior of the NR/SBR matrix nor compromise its intrinsic stability. However, a progressive increase in the maximum degradation rate is observed with increasing GTR content, reaching values close to −12 to −13%/min compared to approximately −10%/min for the composite without GTR. This increase indicates a redistribution of degradation kinetics associated with greater structural heterogeneity [19,20], where domains with different thermal stability, including the continuous elastomeric matrix and the partially crosslinked GTR fraction, coexist within a similar temperature range.

The second stage of thermal decomposition, located between 645 and 665 °C, corresponds to the thermal decomposition of carbon black, which is transformed into CO_2_. With the increase in the GTR content, a slight shift towards lower temperatures (from 665 to 650 °C) and a moderate increase in the decomposition intensity are observed, indicating that the carbon black content in the matrix and that contained in the GTR become clearly referable structures, and are more heterogeneous due mainly to the catalytic effect of the impurities of the carbon black originally used in the waste tires. This behavior is consistent with previous studies where it is observed that at no time does this behavior alter the thermal decomposition phase of the matrix.

From a structural perspective, the thermal results confirm that GTR generated interfacial heterogeneity and partially crosslinked domains in the 10 and 20 phr GTR compounds, which affect the degradation kinetics and carbon evolution without significantly modifying the fundamental thermal stability of the base elastomer [21]. Therefore, the effect of GTR is predominantly structural above 20 phr GTR.

### 3.4. Sanning Electron Micrographs (SEMs)

Scanning electron micrographs have been applied to research to analyze the concentration of reinforcement and morphology. Therefore, as previously mentioned in the “Mechanical Properties” section and as seen in the micrographs in Figure 2b, the presence of amounts greater than 40 phr of GTR allows for a significant decrease in the tensile properties (except the Young’s modulus). This is due to the GTR acting simultaneously as a stiffening phase and as a source of stress concentration, but when the continuity of the elastomeric matrix is broken due to the lack of interaction between the GTR and the matrix, the tensile properties decline significantly.

### 3.5. Dielectric Characterization

#### 3.5.1. Electric Modulus Analysis (M′ and M″)

**Figure 3:**
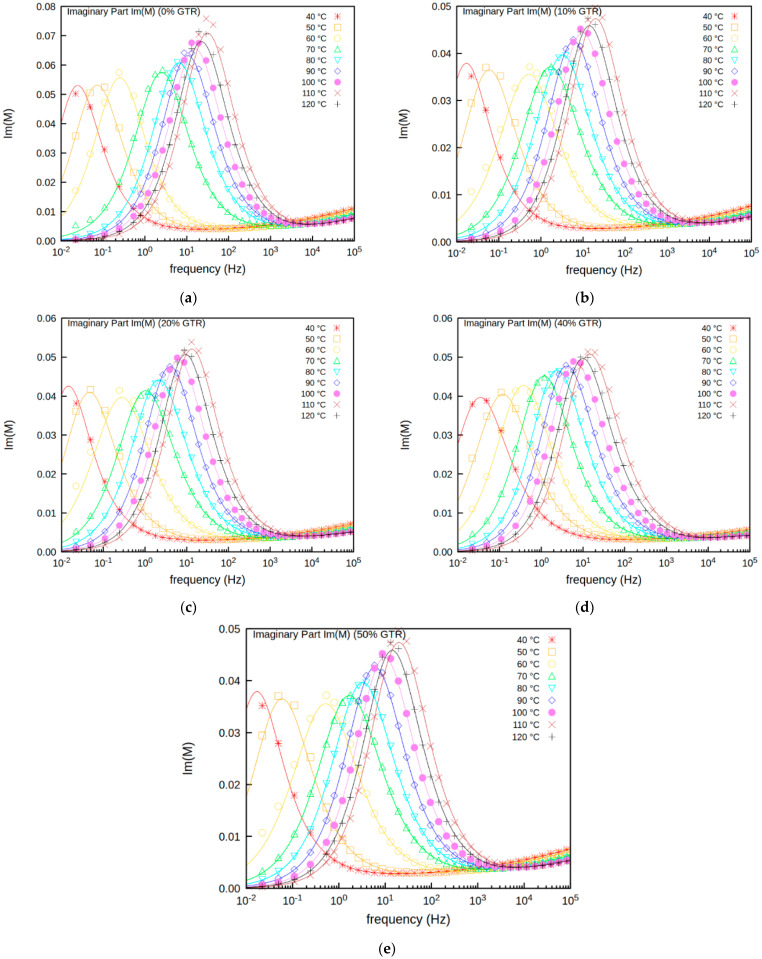
Frequency dependence of the imaginary electric modulus (M″) for NR/SBR–GTR compounds with different recycled contents (0–50 phr) at temperatures between 40 and 120 °C, showing thermally activated relaxation behavior. (**a**) Compounds with 0%GTR, (**b**) compounds with 10%GTR, (**c**) compounds with 20%GTR, (**d**) compounds with 40%GTR, (**e**) compounds with 50%GTR.

**Figure 4:**
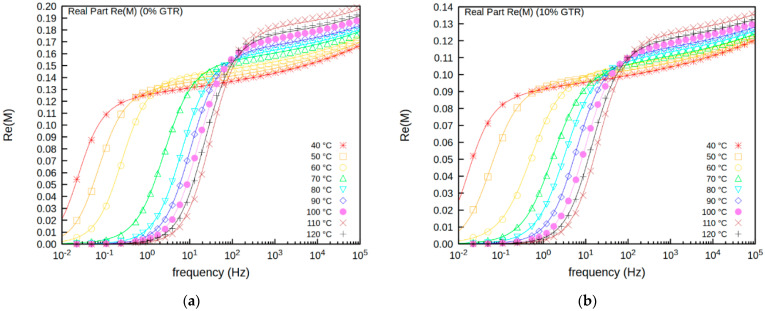

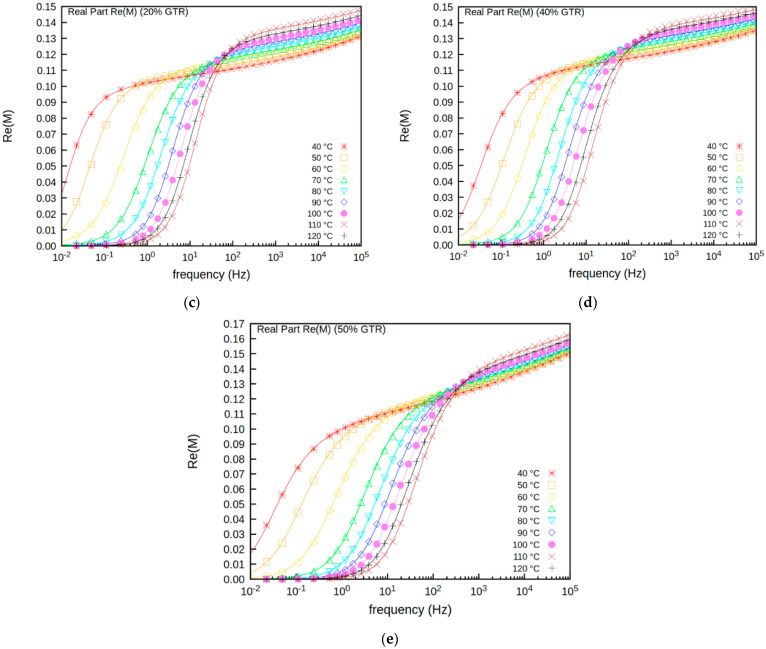
Frequency dependence of the real electric modulus (M′) for NR/SBR–GTR compounds with different recycled contents (0–50 phr) at temperatures between 40 and 120 °C. (**a**) Compounds with 0%GTR, (**b**) compounds with 10%GTR, (**c**) compounds with 20%GTR, (**d**) compounds with 40%GTR, (**e**) compounds with 50%GTR.

The electrical modulus (M′ = 1/ε′), observed in Figure 3 and Figure 4, has been used to analyze the dielectric relaxation processes of NR/SBR/GTR compounds [22,23]. The low-frequency permittivity associated with the polarization of elastomeric compounds allows for a clearer identification of the relaxation phenomena related to the charge in heterogeneous systems.

The imaginary electrical modulus (M″) spectra, observed in Figure 3a–e, shows a single well-defined relaxation peak within the frequency range (10^−2^–10^5^ Hz), indicating the presence of a dominant relaxation process related to conductivity.

For the NR/SBR composite (Figure 3a), the maximum of M″ shifts significantly towards higher frequencies as the temperature increases. At the lowest temperature (40 °C), the relaxation peak is located near 5 × 10^−2^ Hz, with an amplitude of approximately M″ ≈ 0.050–0.055. As the temperature increases, the peak progressively shifts to higher frequencies, reaching values close to 30–60 Hz at 120 °C, where the maximum amplitude increases to approximately M″ ≈ 0.070–0.075.

At higher GTR contents, the relaxation behavior remains qualitatively similar, but the peak becomes slightly broader. For 40 phr GTR (Figure 3d), the maximum of M″ shifts from approximately 0.3–0.5 Hz at low temperature to about 10–25 Hz at high temperature, while for 50 phr GTR (Figure 4e), the peak position evolves from roughly 0.5 Hz to around 10–20 Hz across the same temperature interval. The presence of silica contributes not only to the mechanical reinforcement of the NR/SBR matrix but also to the dielectric response of the composites. Due to its high specific surface area and insulating character, silica promotes the formation of heterogeneous interfaces where charge accumulation occurs, enhancing Maxwell–Wagner–Sillars interfacial polarization.

This systematic shift in the relaxation frequency (Equation (1)) corresponds to a strong reduction in the characteristic relaxation time by two to three orders of magnitude with increasing temperature. This behavior is characteristic of thermally activated charge-movement conduction mechanisms, which are frequently observed in heterogeneous elastomer systems [24,25] containing carbon black or conductive domains originating from GTR.

τ_m_ ≈ 1/(2π · F_max_) (1)

This effect indicates an increase in the heterogeneity of the electrical microstructure as a function of the GTR content. The observed behavior can also be associated with the interfacial Maxwell–Wagner–Sillars (MWS) polarization, which is commonly detected in heterogeneous matrix containing phases with different electrical conductivities and dielectric constants. In the analyzed compounds, the coexistence of the NR/SBR elastomer matrix and partially devulcanized GTR domains introduces numerous internal interfaces where charge carriers can accumulate under an alternating electric field. The effect becomes particularly noticeable at higher GTR contents, where the relaxation peak shifts from approximately 10^−1^ Hz at 40 °C to about 10–30 Hz at 120 °C for formulations containing 40–50 phr GTR (Figure 3d,e). This behavior has also been observed in elastomeric compounds containing GTR [24,25].

The performance of the actual electrical modulus (M′) further supports this interpretation. As shown in Figure 4a–e, M′ remains close to zero at low frequencies due to the dominance of long-range charge migration. With increasing frequency, M′ gradually increases until reaching a high-frequency plateau corresponding to the relaxation regime of the system. For NR/SBR samples (Figure 4a), this transition occurs approximately between 10^0^ and 10^2^ Hz, depending on the temperature. Comparable behavior is observed for the GTR-containing compounds (Figure 4b–e), although the transition region becomes slightly broader as the GTR content increases, which is consistent with the broader distribution of relaxation times already observed in the M″ spectra. Importantly, the incorporation of GTR does not generate additional relaxation peaks in the M″ spectra, even at the highest loading of 50 phr (Figure 4e). This observation indicates that GTR does not introduce new independent dielectric relaxation mechanisms.

It should be noted that, although the total filler content was kept constant across formulations through a proportional reduction in fresh silica and carbon black, the dispersion state of the carbon black fraction differs fundamentally depending on its origin. Freshly incorporated carbon black is distributed throughout the NR/SBR matrix under controlled mixing conditions, yielding a comparatively homogeneous spatial arrangement. In contrast, the carbon black residues present within the GTR particles remain structurally confined within pre-crosslinked rubber domains and cannot redistribute freely during compounding. This confinement generates localized carbon-rich regions that act as discrete interfaces with substantially different electrical conductivity and dielectric permittivity relative to the surrounding elastomeric matrix. As GTR content increases, the spatial density of such heterogeneous interfaces grows, providing additional sites for charge accumulation under an alternating electric field and thereby amplifying the Maxwell–Wagner–Sillars interfacial polarization response. This mechanism is directly responsible for the progressive shift in the M″ relaxation peak toward lower frequencies and for the broadening of the relaxation time distribution observed at higher GTR loadings.

#### 3.5.2. Real and Imaginary Permittivity (ε′ and ε″)

**Figure 5:**
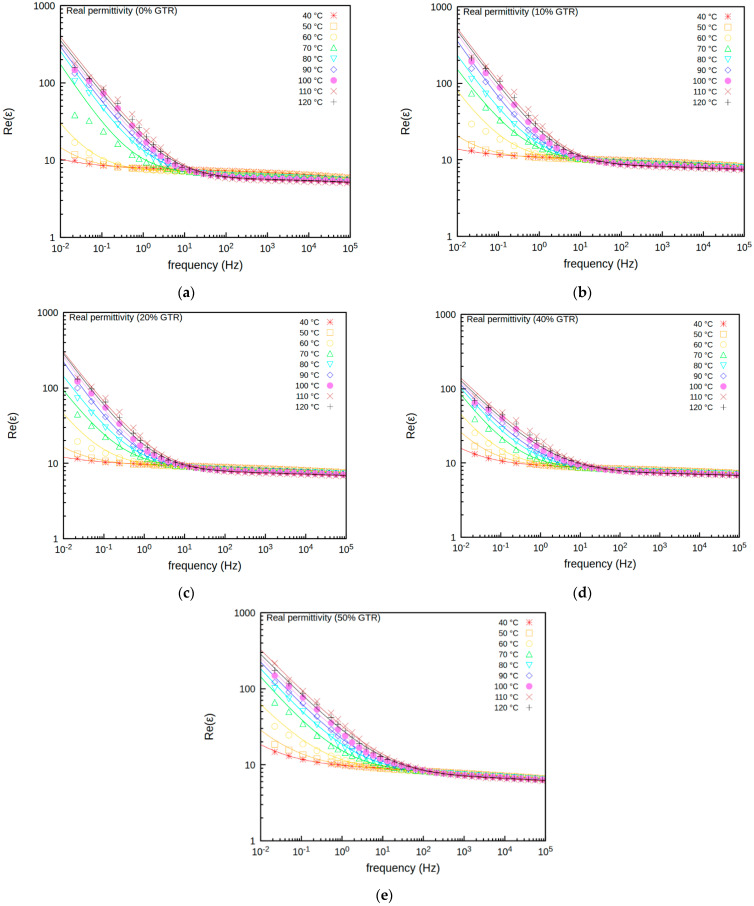
Frequency dependence of the real permittivity (ε′) for NR/SBR–GTR compounds with different recycled contents (0–50 phr) at temperatures between 40 and 120 °C, illustrating interfacial polarization effects. (**a**) Compounds with 0%GTR, (**b**) compounds with 10%GTR, (**c**) compounds with 20%GTR, (**d**) compounds with 40%GTR, (**e**) compounds with 50%GTR.

The real dielectric permittivity (ε′) of the NR/SBR/GTR compounds as a function of frequency and temperature is observed in Figure 5a–e. For all formulations, ε′ decreases with increasing frequency, characteristic of heterogeneous polymer systems in which several polarization mechanisms contribute to the dielectric response.

For the reference sample without GTR (Figure 5a), the permittivity reaches values close to ε′ ≈ 120–150 at 10^−2^ Hz depending on temperature. As frequency increases, ε′ progressively decreases, reaching approximately ε′ ≈ 7–8 at 10^3^ Hz and approaching a quasi-constant plateau of ε′ ≈ 6–7 at 10^5^ Hz (Figure 5a). This behavior reflects the progressive inability of dipolar and interfacial polarization mechanisms to follow the applied alternating electric field at higher frequencies.

A similar trend is observed for the formulations containing GTR (Figure 5b–e), although the magnitude of ε′ at low frequencies slightly increases with increasing GTR content. For the composite containing 10 phr GTR (Figure 5b), ε′ values at 10^−2^ Hz are around 150–170, decreasing to approximately 7–8 at 10^3^ Hz and stabilizing close to 6–7 at 10^5^ Hz. For the 20 phr formulation (Figure 5c), ε′ reaches approximately 170–200 at 10^−2^ Hz, while at high frequencies the values remain in the same range observed for the reference sample, around ε′ ≈ 6–7 at 10^5^ Hz.

At higher GTR contents, the increase in low-frequency permittivity becomes more pronounced. In the case of 40 phr GTR (Figure 5d), ε′ approaches 200–230 at 10^−2^ Hz, while for the compounds containing 50 phr GTR (Figure 5e), the low-frequency permittivity reaches values close to ε′ ≈ 230–260. However, at frequencies above 10^3^–10^4^ Hz, all formulations converge toward similar values around ε′ ≈ 6–8, indicating that the intrinsic polarization of the NR/SBR matrix dominates the dielectric response in this high-frequency region.

The strong increase in ε′ at low frequencies is typical of heterogeneous compounds and is mainly attributed to interfacial polarization phenomena, where charge carriers accumulate at interfaces between phases with different electrical conductivities and dielectric constants. In NR/SBR elastomer, these interfaces are associated with the coexistence of elastomer matrix, partially devulcanized GTR particles, and carbon black residues originating from recycled tire rubber. Such behavior is widely described by Maxwell–Wagner–Sillars interfacial polarization in heterogeneous polymer compounds [26,27]. This charge accumulation indicates that the compatibility between the NR/SBR elastomer matrix and the GTR decreases with increasing GTR content. It is observed that the maximum compatibility is obtained under 20 phr GTR.

**Figure 6:**
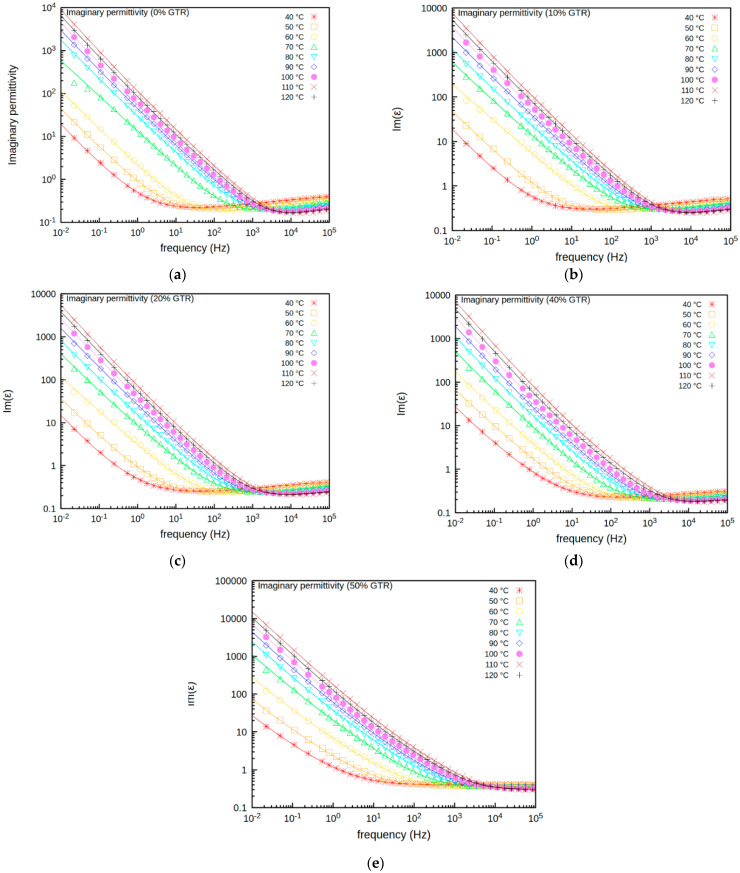
Frequency dependence of the imaginary permittivity (ε″) for NR/SBR–GTR compounds with different recycled contents (0–50 phr) at temperatures between 40 and 120 °C, evidencing conductive and interfacial polarization contributions. (**a**) Compounds with 0%GTR, (**b**) compounds with 10%GTR, (**c**) compounds with 20%GTR, (**d**) compounds with 40%GTR, (**e**) compounds with 50%GTR.

The imaginary dielectric permittivity (ε″), which represents the dielectric loss component associated with energy dissipation processes, is shown in Figure 6a–e. For all formulations, ε″ exhibits a strong decrease with increasing frequency, reflecting the dominant contribution of charge transport and conduction losses at low frequencies.

For the reference compound (Figure 6a), ε″ reaches values of approximately ε″ ≈ 3000–4000 at 10^−2^ Hz, depending on temperature. As frequency increases, the dielectric loss decreases rapidly, reaching approximately ε″ ≈ 10–15 around 10^2^–10^3^ Hz, and approaching values close to ε″ ≈ 3–5 at 10^5^ Hz.

The incorporation of recycled rubber significantly increases the magnitude of ε″ at low frequencies. For the composite containing 10 phr GTR (Figure 6b), the low-frequency dielectric loss reaches approximately **ε**″ ≈ 4000–5000 at 10^−2^ Hz, while for 20 phr GTR (Figure 6c), values close to ε″ ≈ 5000–6000 are observed. At higher GTR loadings, the increase becomes even more pronounced: ε″ ≈ 7000–8000 for 40 phr (Figure 6d) and ε″ ≈ 8000–9000 for 50 phr (Figure 6e) at the lowest frequencies.

Despite these large differences at low frequencies, the dielectric loss values for all formulations tend to converge at higher frequencies. Around 10^3^–10^4^ Hz, ε″ decreases to approximately 2–4 for most compositions (Figure 6a–e), and remains relatively constant up to 10^5^ Hz. This convergence indicates that the high-frequency dielectric behavior is mainly governed by the intrinsic polarization processes of the elastomeric matrix rather than by the conductive pathways introduced by the recycled phase.

The strong low-frequency increase of ε″ observed with increasing GTR content can be attributed to enhanced charge carrier mobility and interfacial charge accumulation within the heterogeneous microstructure. The presence of conductive carbon residues and partially devulcanized rubber domains facilitates charge hopping between localized states, producing the high dielectric losses observed in the low-frequency region. Similar behavior has been reported for elastomer compounds containing recycled tire rubber and conductive fillers, where the dielectric loss is dominated by conduction-related polarization effects [28,29,30].

#### 3.5.3. Electrical Conductivity

##### AC and DC Conductivity Behavior

**Figure 7:**
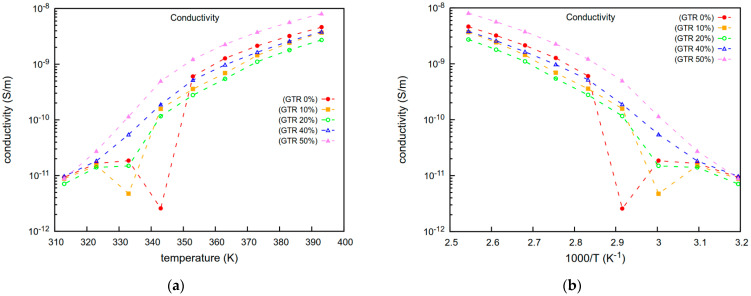
Temperature dependence of DC conductivity (σ_DC_) for NR/SBR–GTR compounds with different recycled contents (0–50 phr). (**a**) Conductivity versus temperature, (**b**) conductivity versus inverse temperature.

The temperature dependence of the electrical conductivity of the NR/SBR/GTR compounds is presented in Figure 7a, where the conductivity values are plotted as a function of temperature for the different GTR contents. The reported values correspond to the dc conductivity (σ_dc_) extracted from the low-frequency plateau of the conductivity spectra. For all formulations, the electrical conductivity increases progressively with temperature, indicating that the charge transport process is thermally activated.

For the reference sample without GTR, the electrical conductivity increases from approximately σ_dc_ ≈ 1 × 10^−10^ S·cm^−1^ at 40 °C to about 3 × 10^−9^ S·cm^−1^ at 120 °C (Figure 7a). The incorporation of GTR slightly increases the conductivity values over the entire temperature range. For the composite containing 10 phr GTR, the conductivity increases from approximately 2 × 10^−10^ S·cm^−1^ at 40 °C to around 5 × 10^−9^ S·cm^−1^ at 120 °C (Figure 7a)**.**

A more noticeable increase is observed for higher GTR amounts. For the formulation containing 20 phr GTR, the conductivity reaches approximately 7 × 10^−9^ S·cm^−1^ at 120 °C (Figure 7a). For 40 phr GTR, σ_dc_ increases from approximately 5 × 10^−10^ S·cm^−1^ at low temperature to nearly 1 × 10^−8^ S·cm^−1^ at 120 °C (Figure 7a), while the compound containing 50 phr GTR reaches conductivity values close to 1–2 × 10^−8^ S·cm^−1^ at the highest temperature (Figure 7a).

Despite this increase, the conductivity values remain within the typical range of insulating elastomer compounds, indicating that no continuous conductive network is formed even at the highest GTR content. This behavior suggests that the conductive pathways associated with the carbon residues present in the GTR remain separated within the elastomer matrix.

The frequency dependence of conductivity in such heterogeneous systems generally follows Jonscher’s universal power law [29], where the total conductivity can be expressed as the sum of a dc component and a frequency-dependent term:

σ(ω) = σ_dc_ + Aω^s^

where σ_dc_ corresponds to the dc conductivity and the second term represents the dispersive contribution associated with localized charge carrier dynamics. Such behavior is typical of disordered polymer systems and elastomer compounds containing conductive residues or filler particles [29,30,31].

##### Arrhenius Analysis and Activation Energy

To analyze the conduction mechanism, the conductivity data were represented in Arrhenius form, plotting the logarithm of conductivity as a function of the inverse absolute temperature, as shown in Figure 7b.

The resulting plots exhibit approximately linear behavior over the investigated temperature range, indicating that the electrical conduction process follows a thermally activated Arrhenius-type mechanism [30,31,32], which can be described by:

$$\sigma =\sigma_{0}\cdot exp\left(\frac{-Ea}{k\cdot T}\right)$$

where **σ_0_** is the pre-exponential factor, Ea is the activation energy for charge transport, k is the Boltzmann constant and T is the absolute temperature.

From the slope of the Arrhenius plots (Figure 7b), activation energies on the order of 0.35–0.45 eV can be estimated for the different formulations. The reference NR/SBR compound shows an activation energy close to Ea ≈ 0.44 eV (Figure 7b). Slightly lower values are observed for the compounds containing GTR. The 10 phr GTR formulation exhibits an activation energy of approximately 0.42 eV, while values close to 0.40 eV and 0.38 eV are estimated for the 20 phr and 40 phr compounds, respectively (Figure 7b). The composite containing 50 phr GTR shows the lowest activation energy, close to 0.36–0.37 eV (Figure 7b).

The decrease in activation energy with increasing GTR content suggests that GTR introduces aggregates that facilitate charge movement between neighboring conductive domains. These domains are mainly associated with carbon black residues and partially devulcanized GTR; however, the presence of SiO_2_ also improves this decrease.

To contextualize the electrical behavior of the 50 phr GTR compound within the broader framework of recycled rubber systems, it is instructive to consider the structural evolution reported by Ismail et al. [33] for recycled rubber powder-filled natural rubber compounds. In that study, progressive incorporation of recycled rubber powder was shown to increase matrix viscosity and stiffness while reducing tensile strength beyond 10 phr, an effect attributed to particle agglomeration and weakened interfacial adhesion. The analogy with our system is relevant: in both cases, the accumulation of pre-crosslinked rubber domains introduces structural discontinuities that modify stress transfer and interfacial architecture. In the present compounds, these discontinuities are additionally associated with conductive carbon black residues confined within the GTR phase, which progressively increase the density of electrically heterogeneous interfaces without forming a macroscopically continuous conductive network. The σdc values measured at 120 °C for the 50 phr GTR formulation (approximately 1–2 × 10^−8^ S·cm^−1^) remain well within the insulating range characteristic of elastomeric matrices and are several orders of magnitude below the conductivity values typically associated with percolated carbon black networks in rubber compounds, which generally exceed 10^−4^ S·cm^−1^. Nevertheless, the sharp increase in imaginary permittivity to values of ε″ ≈ 8000–9000 at low frequencies, combined with the progressive reduction in activation energy to approximately 0.36–0.37 eV, indicates that the system approaches a structural threshold at which inter-domain charge hopping becomes increasingly efficient. This behavior can be associated with a pre-percolation regime in which localized conductive pathways are well developed but remain spatially discontinuous, suggesting that formulations beyond 50 phr GTR could potentially reach or exceed the percolation threshold, with significant consequences for both the dielectric and mechanical performance of the compounds.

##### Conduction Mechanism

The obtained activation energies are consistent with the conduction mechanisms by thermally activated motions [30,31,32], which are observed in polymeric compounds containing dispersed conductive particles or carbonaceous residues. In these systems, charge carriers move through localized states separated by potential barriers, and the probability of movement increases with temperature.

The absence of large changes in conductivity and the relatively moderate increase in σdc with GTR content indicate that the system remains below the threshold of electrical percolation, even at 50 phr. Consequently, the electrical transport in NR/SBR/GTR compounds is mainly governed by charge movements combined with interfacial polarization effects, which is consistent with the dielectric behavior observed previously in the electrical modulus and permittivity analyses [34,35,36].

#### 3.5.4. Sublinear Exponent (*n*)

**Figure 8:**
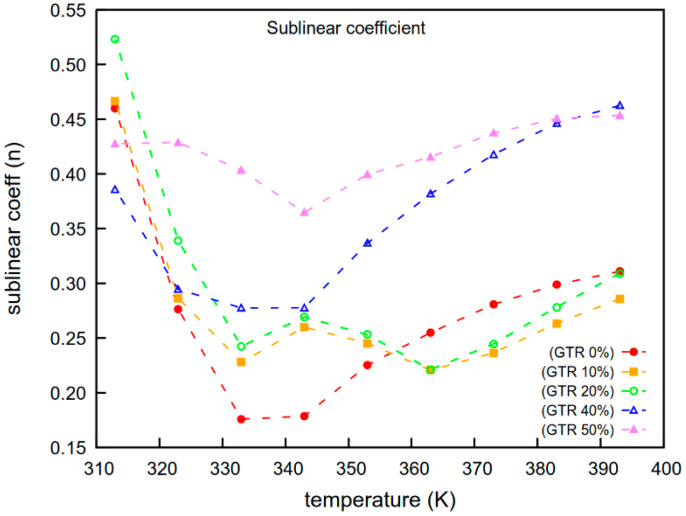
Temperature dependence of the sublinear exponent (*n*) derived from Jonscher’s power law for NR/SBR–GTR compounds with different recycled contents (0–50 phr).

The temperature dependence of the sublinear exponent *n*, obtained from the Jonscher universal power law, is presented in Figure 8 for NR/SBR/GTR compounds with different recycled rubber contents. In disordered dielectric and semiconductor systems, the exponent *n* provides useful information about the degree of interaction between charge carriers and the structural heterogeneity of the medium. In general, values of 0 < *n* < 1 are associated with sublinear dispersive conduction, which is commonly attributed to localized motional transport in structurally disordered materials [33,37,38].

For all compounds, the values of *n* are less than 1 over the entire temperature range, confirming that the AC conductivity follows a sublinear frequency dependence typical of heterogeneous polymer systems. However, the evolution of *n* with temperature is not continuous, which highlights that conduction in heterogeneous elastomeric structures is governed by a combination of temperature-activated charge movements and microstructural constraints imposed by the heterogeneous elastomer–GTR structure.

Compounds with a GTR of 0–20 phr show lower *n* values in the intermediate temperature range, with minima of approximately 0.18–0.24 (Figure 8, 0–20 phr curves), while systems with a GTR of 40–50 phr maintain significantly higher values, typically in the range of 0.37–0.46 at high temperature (Figure 8, 40–50 phr curves). An increase in GTR content tends to increase the *n* value, especially above 353 K, where the 40 and 50 phr curves clearly separate from the formulations with a lower GTR.

This behavior suggests that the incorporation of larger amounts of GTR modifies the dispersive component of charge transport by increasing the number of interfaces and localized conducting regions associated with carbon black residues and partially devulcanized GTR domains. In this context, higher values of *n* can be interpreted as indicative of a less dispersive response and a more effective participation of thermally activated motions in the AC conduction process.

Therefore, the evolution of *n* as a function of temperature is consistent with the analyses of conductivity, permittivity and electrical modulus. This fact allows us to interpret that the electrical response of NR/SBR/GTR compounds is governed by the thermally activated charge movement combined with interfacial polarization effects associated with the heterogeneous microstructure of the GTR [33,37,38].

The characteristics of compounds containing GTR allow for good performance in structural–mechanical applications, as has been demonstrated in other research articles [39,40].

## 4. Conclusions

The influence of devulcanized ground tire rubber (GTR) on the structural, mechanical, thermal, and dielectric properties of NR/SBR compounds was investigated. Thermogravimetric analysis showed that the main degradation temperature of the elastomeric matrix remained essentially unchanged with increasing GTR content, indicating that the thermal stability of the NR/SBR matrix is preserved.

Mechanically, increasing GTR content resulted in a higher elastic modulus, while tensile strength, elongation at break, and toughness decreased, reflecting the increasing influence of matrix–GTR interfacial interactions and stress concentration effects. FTIR and swelling analyses indicated enhanced matrix–GTR interaction at intermediate GTR contents (10–20 phr), suggesting partial integration of the recycled phase within the elastomer network.

Dielectric spectroscopy revealed strong Maxwell–Wagner–Sillars interfacial polarization and thermally activated charge transport. Although electrical conductivity increased moderately with temperature and GTR content, no evidence of a new conduction mechanism or electrical percolation was observed. The electrical response remained governed by charge movement between localized conducting domains associated with the GTR phase.

Overall, the properties of NR/SBR/GTR compounds are mainly controlled by the interfacial structure introduced by GTR. These results provide a basis for the development of sustainable elastomeric materials with high recycled content while maintaining controlled mechanical and dielectric performance.

## Figures and Tables

**Table 1:** Sample codes and formulations of NR/SBR–GTR compounds (phr, per hundred rubber).

Sample Code	NR	SBR	GTR	SiO_2_	CB	S	ZnO	HSt	TBBS	TMTD
0GTRNRSBR	50	50	0	30	30	2	5	3	1	0.25
10GTRNRSBR	48	46	10	29	27	2	5	3	1	0.25
20GTRNRSBR	46	41	20	28	24	2	5	3	1	0.25
40GTRNRSBR	42	34	40	26	18	2	5	3	1	0.25
50GTRNRSBR	40	30	50	25	15	2	5	3	1	0.25

**Table 2:** Mechanical and physical properties of NR/SBR–GTR compounds (mean ± standard deviation).

Sample	Young Modulus (MPa)	Tensile Strength (MPa)	Elongation at Break	Toughness (J)	Hardness (Shore A)	Density (g/cm^3^)	Swelling Degree (%)
0GTRNRSBR	2.01 ± 0.26	18.7 ± 1.65	1051 ± 113	52.3 ± 3.14	49.2 ± 0.6	1.047 ± 0.02	233 ± 13
10GTRNRSBR	2.18 ± 0.13	13.4 ± 1.21	712 ± 61	35.0 ± 2.29	48.7 ± 0.3	1.039 ± 0.01	228 ± 16
20GTRNRSBR	1.93 ± 0.21	12.8 ± 1.01	759 ± 69	29.1 ± 2.01	53.5 ± 1.1	1.051 ± 0.08	231 ± 13
40GTRNRSBR	2.21 ± 0.19	12.5 ± 0.93	703 ± 80	27.6 ± 1.76	58.6 ± 1.9	1.046 ± 0.06	239 ± 14
50GTRNRSBR	2.38 ± 0.10	11.9 ± 0.42	689 ± 56	28.7 ± 1.98	56.9 ± 2.1	1.054 ± 0.05	246 ± 17

**Table 3:** Most significant bands of NR/GTR composites [18,19,20].

Wavenumber (cm^−1^)	Assignments	Component
1537	Zn carboxylic salts	Stearic acid
1463	-CH_2_-stretching	Elastomeric compound
1374	-CH_3_ symmetric bend	Elastomeric compound
1063	Si-O	Oxide silicon
1018	-C-C-	Carbon black
960	Trans group–CH=CH–	Elastomeric compound
797	-C=C-H our of plane	Elastomeric compound
694	CH of the aromatic ring of SBR	Elastomeric compound
453	S-S bonds	Elastomeric compound

## Data Availability

The original contributions presented in this study are included in the article. Further inquiries can be directed to the corresponding author.
